# Whole Blood Gene Expression Profiles in Insulin Resistant Latinos with the Metabolic Syndrome

**DOI:** 10.1371/journal.pone.0084002

**Published:** 2013-12-17

**Authors:** Samantha E. Tangen, Darwin Tsinajinnie, Martha Nuñez, Gabriel Q. Shaibi, Lawrence J. Mandarino, Dawn K. Coletta

**Affiliations:** 1 School of Life Sciences, Arizona State University, Tempe, Arizona, United States of America; 2 College of Nursing & Health Innovation, Arizona State University, Tempe, Arizona, United States of America; 3 Mayo Clinic in Arizona, Scottsdale, Arizona, United States of America; 4 Department of Basic Medical Sciences, University of Arizona College of Medicine – Phoenix, Phoenix, Arizona; Pennington Biomed Research Center, United States of America

## Abstract

Although insulin resistance in skeletal muscle is well-characterized, the role of circulating whole blood in the metabolic syndrome phenotype is not well understood. We set out to test the hypothesis that genes involved in inflammation, insulin signaling and mitochondrial function would be altered in expression in the whole blood of individuals with metabolic syndrome. We further wanted to examine whether similar relationships that we have found previously in skeletal muscle exist in peripheral whole blood cells. All subjects (n=184) were Latino descent from the Arizona Insulin Resistance registry. Subjects were classified based on the metabolic syndrome phenotype according to the National Cholesterol Education Program’s Adult Treatment Panel III. Of the 184 Latino subjects in the study, 74 were classified with the metabolic syndrome and 110 were without. Whole blood gene expression profiling was performed using the Agilent 4x44K Whole Human Genome Microarray. Whole blood microarray analysis identified 1,432 probes that were altered in expression ≥1.2 fold and P<0.05 after Benjamini-Hochberg in the metabolic syndrome subjects. KEGG pathway analysis revealed significant enrichment for pathways including ribosome, oxidative phosphorylation and MAPK signaling (all Benjamini-Hochberg P<0.05). Whole blood mRNA expression changes observed in the microarray data were confirmed by quantitative RT-PCR. Transcription factor binding motif enrichment analysis revealed E2F1, ELK1, NF-kappaB, STAT1 and STAT3 significantly enriched after Bonferroni correction (all P<0.05). The results of the present study demonstrate that whole blood is a useful tissue for studying the metabolic syndrome and its underlying insulin resistance although the relationship between blood and skeletal muscle differs.

## Introduction

The metabolic syndrome is a complex pathological state that is associated with obesity, hypertension, atherosclerotic cardiovascular disease, and type 2 diabetes [1]. An underlying feature that is present across these common diseases is insulin resistance, which is defined as a decreased ability of insulin to perform its biological functions. Moreover, the pathophysiology of the metabolic syndrome and its associated diseases are attributable to a low grade inflammation [2]. Over the past three decades, the prevalence of the metabolic syndrome has increased, largely due to the increased prevalence observed for obesity and type 2 diabetes [3-6]. The metabolic syndrome (also referred to as insulin resistance syndrome and syndrome X), can be defined in various ways [7], with the essential components including obesity, dyslipidemia, hypertension, and glucose intolerance. 

To date, the majority of our work has focused on skeletal muscle insulin resistance, where we have used global proteomics and transcriptomic approaches to demonstrate the relationship between inflammation, extracellular remodeling, cytoskeletal interactions, and mitochondrial function [2]. Moreover, many cellular, molecular and biochemical defects have been shown to contribute to the pathophysiology of insulin resistance including impaired insulin signaling, reduced insulin-stimulated glucose uptake, lower insulin-stimulated activities of enzymes such as hexokinase and glycogen synthase, increased toxic lipid metabolites and impaired mitochondrial function [8-16]. Studying skeletal muscle in relation to insulin resistance and its associated diseases such as the metabolic syndrome is particularly important, as under normal physiological conditions this tissue is the major site of insulin-stimulated glucose disposal [17]. To date, the role of peripheral whole blood in the metabolic syndrome and the underlying insulin resistance is less understood. As such, one of the main objectives of this study was to determine whether relationships that we have found in the skeletal muscle similarly exist in the peripheral whole blood cells, so that we could eventually use whole blood as a surrogate tissue for studying the metabolic syndrome.

By using global transcriptomic approaches, we set out to test the hypothesis that there would be changes in the expression of genes involved in inflammation, insulin signaling and mitochondrial function in the whole blood of the subjects classified with the metabolic syndrome. Conducting this study using global gene expression profiling allowed us to test these hypotheses and at the same time identify novel targets of metabolic syndrome and its underlying insulin resistance in whole blood, which could allow for development of new strategies for diagnosis as well as the identification of novel treatment targets. 

## Methods

### Subjects

All subjects were Latino descent and were participants of a community-based diabetes registry. Subjects reported to the Clinical Research Unit at Arizona State University following an overnight fast. Metabolic, anthropometric, demographic, and medical history information were obtained on all subjects. The study protocol was approved by the Institutional Review Board of the Arizona State University and all participants provided informed written consent prior to enrollment. 

### Classification of metabolic syndrome

Subjects in the present study were classified based on the metabolic syndrome phenotype according to the National Cholesterol Education Program’s Adult Treatment Panel III (NCEP: ATP III). When 3 out of the 5 following characteristics were present, a diagnosis of metabolic syndrome was determined: (1) abdominal obesity, given as waist circumference (> 102 cm men/> 88 cm women), (2) HDL cholesterol (

< 40 mg/dl men/< 50 mg/dl in women), (3) blood pressure ≥ 130/ ≥85 mmHg, (4) fasting glucose ≥ 110 mg/dl and (5) triglycerides ≥ 150 mg/dl

### Oral glucose tolerance test (OGTT)

A 75-g OGTT was performed on each participant following a 10-12 hour overnight fast as previously described [18]. Since insulin resistance is a unifying hypothesis to describe the pathophysiology of the metabolic syndrome [19,20], we also calculated the Matsuda Index of each subject in the study [21]. 

### Whole blood processing for RNA isolation

Blood was collected into PAXgene Blood RNA tubes (BD Diagnostics, Franklin Lakes, NJ) and stored at -80°C until processed. Total RNA was isolated using the PAXgene Blood RNA Kit, as per the manufacturer’s instructions (Qiagen, Valencia, CA). RNA quality and quantity were determined using capillary electrophoresis on an Agilent 2100 Bioanalyzer automated analysis system (Agilent Technologies, Palo Alto, CA). The RNA absorbance ratio (A_260_/A_280_) values and RNA integrity number (RIN) values were 2.1 ± 0.002 and 8.8 ± 0.03, respectively. 

### Microarray processing

Total RNA (500 ng) was amplified and labeled using the Amino Allyl MessageAmp™ II aRNA Amplification Kit, as per manufacturer’s instructions (Life Technologies, Carlsbad, CA). After labeling, antisense RNA (aRNA) was fragmented using Agilent Gene Expression Hybridization Kit (Agilent Technologies, Palo Alto, CA), as per instructions. The fragmented aRNA was hybridized to the 4x44K Whole Human Genome Microarray (Agilent Technologies, Palo Alto, CA) using a SureHyb DNA Microarray Hybridization Chamber at 65°C, for 17 hours in a rotating incubator. After hybridization, slides were washed in Gene Expression wash buffers 1 and 2 as per instructions, and then scanned with an Agilent DNA microarray scanner (Agilent Technologies, Palo Alto, CA). 

### Microarray analysis

**Figure 1:**
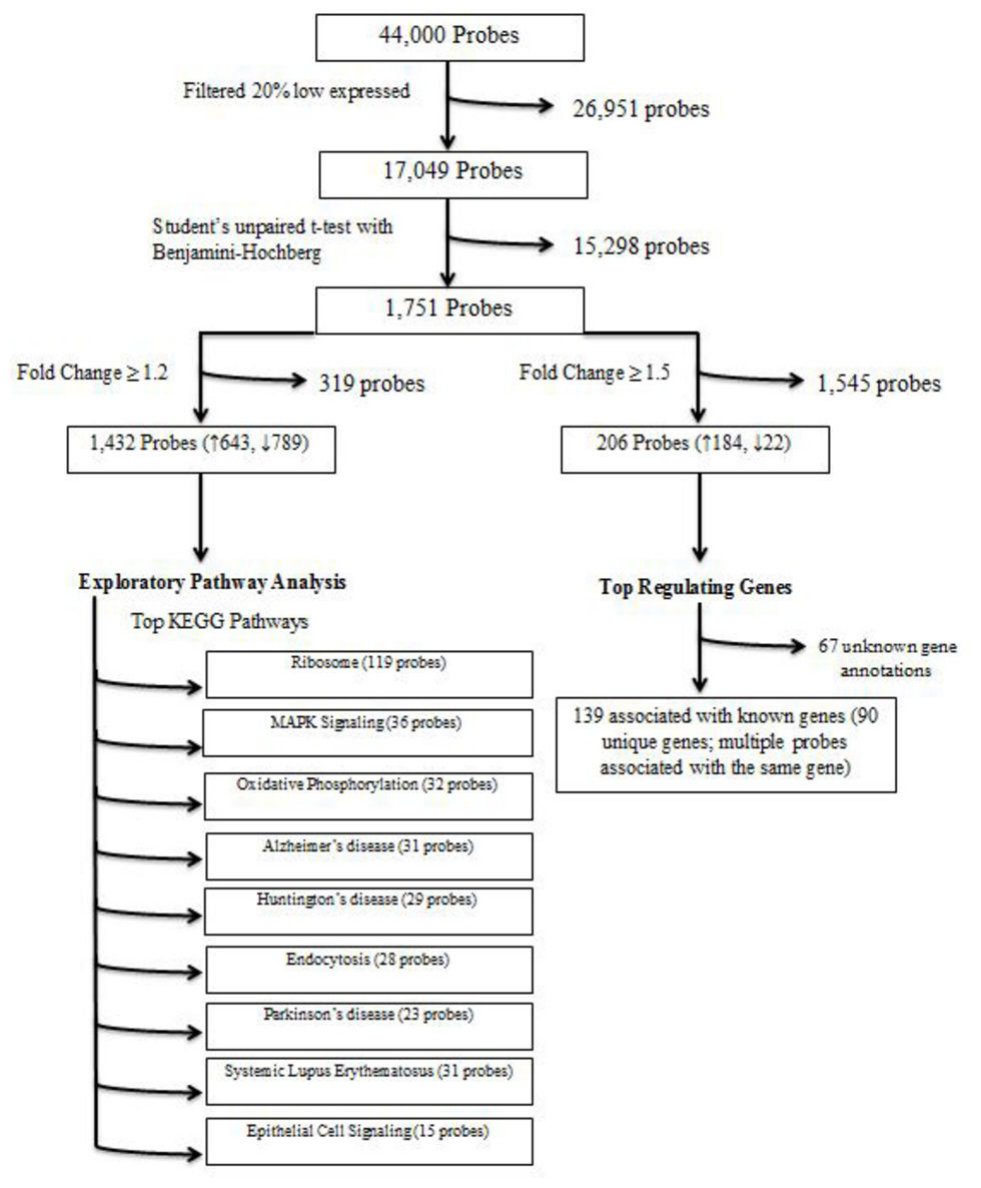
Flow diagram of the steps used in the analysis of the microarray data.

Feature Extraction Software version 10.5.1.1 (Agilent Technologies, Palo Alto, CA), was used for analysis of the array images. The data generated from the image analysis has been deposited in the ArrayExpress database (www.ebi.ac.uk/arrayexpress) under accession number E-MEXP-12345. The data files were imported into GeneSpring version 12 (Agilent Technologies, Palo Alto, CA). Data were log transformed, quantile normalized and baseline to median of all samples. The probes below 20% expression were excluded from the analysis. A detailed overview of the analysis is shown in Figure 1. Expression values obtained were submitted to a Student’s unpaired t-test, and P values were adjusted using the Benjamini-Hochberg multiple testing correction. Significant probes with a Benjamini-Hochberg P < 0.05 and fold change ≥ 1.2 were used for exploratory KEGG pathway analysis using the database for annotation, visualization and integrated discovery (DAVID) (http://david.niaid.nih.gov), as previously described [22]. Moreover, to identify the genes that differed most robustly, we filtered the probe list further to identify probes with a Benjamini-Hochberg P < 0.05 and fold change ≥ 1.5. 

### Transcription factor binding motif enrichment analysis

Overrepresentation of transcription factor binding sites analysis of the differentially expressed genes was performed using Pscan version 1.2.2 [23]. We selected a region 950 base pairs upstream and 50 base pairs (-950 to +50 base pair) downstream of the translational start site for analysis using the JASPAR [24] and TRANSFAC [25] databases. 

### Quantitative Real Time PCR (Q-RT-PCR)

Blood expression of various genes was determined using Q-RT-PCR on the ABI PRISM 7900HT sequence detection system (Life Technologies, Carlsbad, CA). TaqMan Universal Fast PCR master mix reagents and the Assay-On-Demand gene expression primer pair and probes (Life Technologies, Carlsbad, CA) were added to 20 ng cDNA, which was synthesized using the ABI High Capacity cDNA Reverse Transcription Kit, as per manufacturer’s instructions. The quantity of the gene of interest in each sample was normalized to that of GAPDH using the comparative (2^-∆∆^CT) method [26]. Statistical comparisons were performed using Student’s unpaired t-test. 

### Substrate and hormone determinations

Plasma glucose concentration was determined by the glucose oxidase method on an YSI 2300 STAT plus (YSI INC., Yellow Springs, OH). Plasma insulin was measured by enzyme-linked immunosorbent assay (ALPCO Diagnostics, Salem, NH). A comprehensive metabolic panel, lipid panel and hemogram panel were performed by a Clinical Laboratory Improvement Amendments (CLIA) certified commercial laboratory (Sonora Quest Laboratories, Phoenix, AZ). 

### Statistical analysis

Independent sample t-tests and χ^2^ analyses were used to compare characteristics between the Met Syn-YES group and Met Syn-NO group. Additional comparison analyses by sex were performed to examine the differences in phenotypic characteristics within each Met Syn group. Data that did not meet the assumptions for normality were log_10_ transformed for significance; however, untransformed data was presented for ease of interpretation. Analysis of covariance (ANCOVA) was used to adjust for the effects of age, sex, and the interaction between age and sex. Data were expressed as the means ± SE. All data were analyzed using SPSS version 20.0 with a significant level set at P ≤ 0.05. Microarray statistical analysis is described above. 

## Results

### Subjects

The characteristics of the subjects that participated in this study are shown in Table 1. As described above, subjects were categorized into one of two groups based on the NCEP: ATP III metabolic syndrome criteria. Of the 184 in the present study, 74 met the criteria for metabolic syndrome (Met Syn-YES) and 110 subjects were classified as not having the metabolic syndrome (Met Syn-NO). Subjects were matched for sex across the two groups. The subjects in the Met Syn-NO group were slightly younger that the Met Syn-Yes group (35 ± 1 and 39 ± 1, respectively). As shown in Table 1, the subjects categorized in the Met Syn-YES group were obese, dyslipidemic, glucose intolerant and had elevated blood pressure. Moreover, the subjects in the Met Syn-YES group were more insulin resistant than the Met Syn-NO group as measured by the Matsuda Index. All these differences remained significant after adjusting for potential covariates including age, sex, and the interaction between age and sex (Table 1). Further separation by sex for both Met Syn-YES and Met Syn-NO, as shown in Table S1, exhibited significant differences in body fat percentage, HDL, ALT, and 2 hour plasma insulin concentration. 

**Table 1:** Characteristics of subjects classified into one of two groups based on metabolic syndrome criteria.

	**Met Syn-NO**	**Met Syn-YES**	**P Value**	P value by adjustment of age, sex, age*sex
**Gender**	36M/74F	25M/49F	NS	-
**Age (years)**	35 ± 1	39 ± 1	<0.01	**-**
**Body Mass Index (kg/m^2^)**	28.3 ± 0.5	35.1 ± 1.1	<0.0001	<0.0001
**Body Fat (%)**	28.9 ± 0.7	34.5 ± 0.9	<0.0001	<0.0001
**Systolic Blood Pressure (mmHg)**	114.7 ± 1.1	128.4 ± 2.4	<0.0001	<0.0001
**Diastolic Blood Pressure (mmHg)**	75.3 ± 0.8	81.5 ± 1.2	<0.0001	<0.0001
**Hip Circumference (cm)**	104.9 ± 1.0	116.2 ± 1.5	<0.0001	<0.0001
**Waist Circumference (cm)**	95.1 ± 1.2	109.1 ± 1.7	<0.0001	<0.0001
**Triglyceride (mg/dL)**	107.7 ± 4.3	204.7 ± 12.2	<0.0001	<0.0001
**Cholesterol (mg/dL)**	166.9 ± 2.9	191.0 ± 4.7	<0.0001	<0.001
**High Density Lipoprotein (mg/dL)**	47.8 ± 1.1	37.6 ± 1.0	<0.0001	<0.0001
**Low Density Lipoprotein (mg/dL)**	102.4 ± 2.5	117.4 ± 3.9	<0.001	<0.01
**Very Low Density Lipoprotein (mg/dL)**	18.0 ± 0.7	30.9 ± 1.5	<0.0001	<0.0001
**Alanine Aminotransferase (IU/L)**	22.9 ± 1.3	41.9 ± 3.6	<0.0001	<0.0001
**Aspartate Aminotransferase (IU/L)**	22.9 ± 0.9	30.9 ± 2.0	<0.0001	<0.001
**Fasting Plasma Glucose (mg/dL)**	92.5 ± 1.8	112.8 ± 4.8	<0.0001	<0.0001
**2 Hour Plasma Glucose (mg/dL)**	124.0 ± 4.6	181.4 ± 9.4	<0.0001	<0.0001
**Hemoglobin AIc (%)**	5.6 ± 0.1	6.2 ± 0.1	<0.0001	<0.0001
**Fasting Plasma Insulin (uIU/mL)**	7.3 ± 0.5	12.8 ± 1.3	<0.0001	<0.0001
**2 Hour Plasma Insulin (uIU/mL)**	59.6 ± 5.4	104.9 ± 12.8	<0.0001	<0.0001
**Matsuda Index**	7.3 ± 0.7	3.5 ± 0.4	<0.0001	<0.0001

Data are mean ± SE

### Analysis of microarray data

Microarray analysis was performed on RNA isolated from whole blood obtained from the 184 subjects who participated in this study. Expression values were normalized as described above then subjected to Student’s unpaired t-test, and P values were adjusted using the Benjamini-Hochberg multiple testing correction. Figure 1 provides an overview of the steps used in the analysis of the microarray data. 

A total of 1,432 probes were altered in expression ≥ 1.2 fold and P < 0.05 after Benjamini-Hochberg correction. The 1,432 probes were submitted to DAVID for exploratory pathway analysis, and the significant KEGG pathway results are shown in Table 2. The KEGG pathways that were identified from the pathway analysis were ribosome, oxidative phosphorylation, Alzheimer disease, epithelial cell signaling, Huntington disease, systemic lupus erythematosus, Parkinson disease, endocytosis and MAPK signaling (Table S2). Figures S1 and S2 show the fold change data for the individual oxidative phosphorylation and MAPK signaling genes that were identified from the pathway analysis. 

**Table 2:** Results of the significant KEGG pathway analysis performed in DAVID on the 1,432 probes that were altered in expression ≥ 1.2 fold and P < 0.05 after Benjamini-Hochberg correction.

**KEGG Pathway**	**Probe Count**	**Up Regulated**	**Down Regulated**	**Significance**
Ribosome	119	119	0	<0.0001
Oxidative Phosphorylation	32	28	4	<0.0001
Alzheimer Disease	31	22	9	<0.01
Epithelial Cell Signaling	15	1	14	<0.01
Huntington Disease	29	23	6	<0.05
Systemic Lupus Erythematosus	31	1	30	<0.05
Parkinson Disease	23	22	1	<0.05
Endocytosis	28	0	28	≤0.05
MAPK Signaling	36	0	36	0.057

To identify the genes that differed most robustly, we filtered the probe list further to identify probes with a Benjamini-Hochberg P < 0.05 and fold change ≥ 1.5. The filtering analysis revealed 206 probes that were altered in expression. Of the 206 probes, 67 had unknown gene annotations. The remaining 139 probes represented 90 unique genes (there were multiple probes associated with the same gene). Of the 90 unique genes, 20 were lower in expression and the remaining 70 were higher in expression in the Met Syn-YES group as shown in Table 3 and 4, respectively. The most robustly expressed gene that was lower in expression in the Met Syn-YES group was solute carrier family 45, member 3 (SLC45A3). In addition, the gene analysis identified multiple ribosomal proteins, oxidative phosphorylation genes and lactotransferrin (LTF) that were higher in expression in the Met Syn-YES group (Table 4).

**Table 3:** Results of GeneSpring analysis revealed 206 probes that were altered in expression ≥ 1.5 fold and P < 0.05 (Benjamini corrected).

**Probe Name**	**Gene Description**	**Gene Symbol**	**Fold Change**
A_24_P208345	solute carrier family 45, member 3	SLC45A3	-2
A_23_P10559	apoptosis-associated tyrosine kinase	AATK	-1.6
A_23_P501933	calcium channel, voltage-dependent, gamma subunit 6	CACNG6	-1.6
A_23_P166297	ATP-binding cassette, sub-family G, member 1	ABCG1	-1.5
A_23_P127948	adrenomedullin	ADM	-1.5
A_23_P417383	aspartic peptidase, retroviral-like 1	ASPRV1	-1.5
A_23_P4662	B-cell CLL/lymphoma 3	BCL3	-1.5
A_23_P359052	biorientation of chromosomes in cell division 1-like	BOD1L	-1.5
A_23_P210811	CD93 molecule	CD93	-1.5
A_23_P119562	complement factor D (adipsin)	CFD	-1.5
A_23_P7144	chemokine (C-X-C motif) ligand 1 (melanoma growth stimulating activity, alpha)	CXCL1	-1.5
A_23_P257924	v-ets erythroblastosis virus E26 oncogene homolog 2	ETS2	-1.5
A_23_P99661	hypothetical protein FLJ10357	FLJ10357	-1.5
A_23_P67847	UDP-N-acetyl-alpha-D-galactosamine:polypeptide N-acetylgalactosaminyltransferase 14	GALNT14	-1.5
A_23_P110022	GATA binding protein 2	GATA2	-1.5
A_32_P217750	interleukin 3 receptor, alpha (low affinity)	IL3RA	-1.5
A_24_P282060	hypothetical protein LOC100128439	LOC100128439	-1.5
A_23_P114466	transducin (beta)-like 1Y-linked	TBL 1Y	-1.5
A_23_P145965	tyrosylprotein sulfotransferase 1	TPST1	-1.5
A_23_P55606	zinc finger protein 516	ZNF516	-1.5

Of the 206 probes, 67 had unknown gene annotations and the remaining 139 probes represented 90 unique genes (multiple probes associated with the same gene). Of the 90 unique genes, 20 were lower in expression in the Met Syn-YES group.

**Table 4:** Results of GeneSpring analysis revealed 206 probes that were altered in expression ≥ 1.5 fold and P < 0.05 (Benjamini corrected).

**Probe Name**	**Gene Description**	**Gene Symbol**	**Fold Change**
A_32_P220127	ribosomal protein L34	RPL34	+2.7
A_24_P6975	ribosomal protein L34 pseudogene 34	RPL34P34	+2.2
A_32_P31182	ribosomal protein L7 pseudogene 26	RPL7	+2.2
A_32_P196483	ribosomal protein S3A pseudogene 5	RPS3A	+2.2
A_24_P152753	ribosomal protein L31 pseudogene 18	RPL31P18	+2.1
A_24_P41551	ribosomal protein L31 pseudogene 39	RPL31P39	+2.1
A_23_P159650	cytochrome c oxidase subunit VIIb	COX7B	+2.0
A_23_P128192	prefoldin subunit 5	PFDN5	+2.0
A_24_P366546	ribosomal protein L31 pseudogene 10	RPL31P10	+2.0
A_32_P145153	ribosomal protein L31 pseudogene 49	RPL31	+2.0
A_24_P76358	ribosomal protein S3a pseudogene 36	RPS3AP36	+2.0
A_23_P166848	lactotransferrin	LTF	+1.9
A_23_P34018	ribosomal protein L39 pseudogene 10	RPL39	+1.9
A_24_P232856	ribosomal protein L9	RPL9	+1.9
A_24_P186944	ribosomal protein L9 pseudogene 18	RPL9P18	+1.9
A_24_P144666	ribosomal protein S3a pseudogene 10	RPS3AP10	+1.9
A_32_P21384	ribosomal protein L17 pseudogene 22	RPL17	+1.8
A_24_P366165	ribosomal protein L26 pseudogene 12	RPL26P12	+1.8
A_24_P135551	ribosomal protein L26 pseudogene 13	RPL26P13	+1.8
A_24_P212864	ribosomal protein L26 pseudogene 21	RPL26P21	+1.8
A_24_P161914	ribosomal protein L7 pseudogene 13	RPL7P13	+1.8
A_24_P169378	ribosomal protein S7	RPS7	+1.8
A_24_P15765	ribosomal protein S7 pseudogene 5	RPS7P5	+1.8
A_24_P127181	ribosomal protein S7 pseudogene 8	RPS7P8	+1.8
A_23_P168916	carbonic anhydrase I	CA1	+1.7
A_23_P132863	LSM3 homolog, U6 small nuclear RNA associated (S. cerevisiae)	LSM3	+1.7
A_32_P30710	ribosomal protein L23 pseudogene 6	RPL23	+1.7
A_32_P22539	ribosomal protein L26 pseudogene 32	RPL26P32	+1.7
A_23_P33045	ribosomal protein L26 pseudogene 33	RPL26	+1.7
A_24_P106306	ribosomal protein L26-like 1	RPL26L1	+1.7
A_32_P136319	ribosomal protein L36a pseudogene 51	RPL36A	+1.7
A_24_P153324	ribosomal protein L7 pseudogene 44	RPL7P44	+1.7
A_24_P349636	ribosomal protein L7 pseudogene 48	RPL7P48	+1.7
A_24_P84808	ribosomal protein L7 pseudogene 50	RPL7P50	+1.7
A_24_P221366	ribosomal protein S15a pseudogene 17	RPS15A	+1.7
A_24_P375849	ribosomal protein S17 pseudogene 2	RPS17P2	+1.7
A_23_P1206	ribosomal protein S24	RPS24	+1.7
A_32_P4532	ribosomal protein S3a pseudogene 20	RPS3AP20	+1.7
A_23_P59921	SUB1 homolog (S. cerevisiae)	SUB1	+1.7
A_32_P98348	zinc finger protein 525	ZNF525	+1.7
A_23_P170233	cystatin A (stefin A)	CSTA	+1.6
A_23_P8900	cytochrome c oxidase subunit VIc	COX6C	+1.6
A_23_P110811	cytochrome c oxidase subunit VIIc	COX7C	+1.6
A_32_P336445	histidine triad nucleotide binding protein 1	HINT1	+1.6
A_32_P25253	iron-sulfur cluster assembly 1 homolog (S. cerevisiae)	ISCA1	+1.6
A_23_P145777	NADH dehydrogenase (ubiquinone) 1 alpha subcomplex, 4, 9kDa	NDUFA4	+1.6
A_23_P65466	RAB2B, member RAS oncogene family	RAB2B	+1.6
A_23_P65768	ribosomal L24 domain containing 1	RSL24D1	+1.6
A_32_P203154	ribosomal protein L21 pseudogene 134	RPL21	+1.6
A_23_P143958	ribosomal protein L22-like 1	RPL22L1	+1.6
A_23_P128067	ribosomal protein L41	RPL41	+1.6
A_23_P258108	ribosomal protein L9 pseudogene 16	RPL9P16	+1.6
A_23_P14734	ribosomal protein S27-like	RPS27L	+1.6
A_23_P70843	2,3-bisphosphoglycerate mutase	BPGM	+1.5
A_24_P188116	ankyrin repeat domain 2 (stretch responsive muscle)	ANKRD2	+1.5
A_23_P255827	apoptosis inhibitor	FKSG2	+1.5
A_32_P123771	ATP synthase, H+ transporting, mitochondrial F1 complex, epsilon subunit	ATP5E	+1.5
A_23_P138985	CD3d molecule, delta (CD3-TCR complex)	CD3D	+1.5
A_23_P205281	chromosome 14 open reading frame 2	C14orf2	+1.5
A_23_P212854	glycophorin E; glycophorin B (MNS blood group)	GYPB	+1.5
A_23_P413796	HAUS augmin-like complex, subunit 1	HAUS1	+1.5
A_23_P87346	hemoglobin, delta	HBD	+1.5
A_24_P203827	histidine triad nucleotide binding protein 1 pseudogene	HINT1P1	+1.5
A_23_P502425	mitochondrial ribosomal protein L47	MRPL47	+1.5
A_24_P168416	peroxiredoxin 2	PRDX2	+1.5
A_32_P34201	ribosomal protein L6 pseudogene 13	RPL6P13	+1.5
A_24_P575336	ribosomal protein L6 pseudogene 3	RPL6P3	+1.5
A_23_P76961	ribosomal protein S29 pseudogene 11	RPS29	+1.5
A_23_P434809	S100 calcium binding protein A8	S100A8	+1.5
A_23_P93844	translocase of outer mitochondrial membrane 7 homolog (yeast)	TOMM7	+1.5

Of the 206 probes, 67 had unknown gene annotations and the remaining 139 probes represented 90 unique genes (multiple probes associated with the same gene). Of the 90 unique genes, 70 were higher in expression in the Met Syn-YES group.

### Transcription factor binding motif enrichment analysis

To further understand the genes altered in expression in this study, we analyzed the promoter regions (-950 to +50 base pair) of the unique genes that differed most robustly (n=90) for enrichment in transcription factor response element binding motifs using PScan. We used response element profiles available in both the JASPAR and TRANSFAC databases and searched for enrichment that was significant using both databases. Table 5 shows the results of the transcription factor response element binding motifs. 

**Table 5:** Results of the transcription factor binding motif enrichment analysis.

**Transcription Factor**	P Value*
E2F1	<0.01
ELK1	<0.05
NF-kappaB	<0.01
STAT1	<0.001
STAT3	<0.01

^*^ Bonferroni P values refer to enrichment of transcription factors relative to random selection of 5’UTRs in genome (PScan)

### Quantitative RT-PCR analysis to confirm microarray data

To validate the microarray data, we quantitated the changes in mRNA levels of 11 genes (9 of which higher and 2 of which lower in expression using microarray analysis) using RT-PCR in 30 subjects; 15 subjects from Met Syn-YES, and 15 subjects from Met Syn-NO. Subjects in each group were randomly selected from the 184 individuals that participated in the microarray study. Genes for confirmation by RT-PCR were randomly selected from the differentially expressed list. Table 6 shows the results of the RT-PCR analysis. The changes in gene expression determined by Q-RT-PCR were concordant with changes in gene expression determined by microarray analysis.

**Table 6:** Results of the mRNA expression determined by Q-RT-PCR (with corresponding microarray results shown).

**Gene**	**Q-RT-PCR Fold Change**	**Microarray Fold Change**
ATP5E	1.2	1.5
CACNG6	-1.5*	-1.6
COX7B	1.8*	2.0
COX7C	1.8*	1.6
CSTA	1.4*	1.6**^*a*^**
LTF	2.3*	1.9
NDUFA4	1.2*	1.6**^*a*^**
PFDN5	1.5*	2.0**^*a*^**
RPL34	1.6*	2.7**^*a*^**
RPS3A	1.3*	2.2**^*a*^**
SLC45A3	-2.6*	-2

^a^ More than one probe set was significantly altered for this gene on the microarray. The probe set with the greatest fold change in the microarray analysis is represented here. Significance for the genes selected for Q-RT-PCR was assessed looking at each dataset separately and using Student’s unpaired *t*-test. *P < 0.05 vs. control GAPDH RNA (used for Q-RT-PCR analysis).

## Discussion

The present study was undertaken to decipher the role of peripheral whole blood in relation to the metabolic syndrome phenotype. We set out to test the hypothesis that genes involved in inflammation, insulin signaling and mitochondrial function would be altered in expression in the whole blood of individuals with metabolic syndrome. We further wanted to examine whether similar relationships that we have found previously in skeletal muscle exist in peripheral whole blood cells. It has been shown elsewhere that over 80% of the genes expressed in nine different human tissue types overlapped with the peripheral blood transcriptome [27]. Therefore, the peripheral blood cells express a large proportion of the genes in the human genome, suggesting that whole blood may be useful as a surrogate for studying metabolic syndrome and related conditions such as insulin resistance, diabetes and obesity. 

We performed global transcriptomic analyses of peripheral whole blood as it is an easy tissue to obtain and it is therefore non-invasive. We performed genome-wide gene expression profiling on RNA obtained directly from whole blood collected in PAXgene tubes rather than use peripheral blood mononuclear cells (PBMCs). The disadvantage of using the PAXgene tubes and studying whole blood is that the results generated from this gene expression profiling study represent the contribution of RNA from all blood cell types. Whole blood is a complex tissue comprised of many cell types including neutrophils, eosinophils, basophils, B cells, T cells, and monocytes. As such, when using the PAXgene tube approach we cannot definitely state which cell type is contributing the mRNA expression signal. However, the goal for this study was to develop a transcriptome for studying metabolic syndrome in whole blood, and we chose the PAXgene tubes to minimize RNA degradation, to minimize handling and potential activation of blood cells, and to minimize loss of sample in purification of specific sub populations of leukocytes, all of which could affect gene expression levels. 

In the present study, fold changes ranged from -2 to +2.7, which were much lower than our previous studies in skeletal muscle where observed changes range from -3 to +8 [22,28]. Moreover, it has been observed elsewhere that the gene expression levels from PAXgene RNA are much lower when compared to studies using PBMCs [29,30]. Ghosh et al., addressed this issue and suggested that the lower fold change ranges in PAXgene analyses is attributable to the high abundance of globin in the whole blood [30]. Although 70% of whole blood mRNA is represented by globin, we were able to validate the gene expression changes captured in the microarray with quantitative RT-PCR. This indicates that the changes observed in the present study were present, suggesting that the genes and pathways are biologically relevant and could be used for development of new strategies for diagnosis as well as the identification of novel treatment targets. 

In this study, each individual was classified based on metabolic syndrome criteria. Metabolic syndrome was used to classify our sample population due to its known concordance with type 2 diabetes, obesity, hypertension, and atherosclerotic cardiovascular disease [1]. For this study, the relationship between insulin resistance and the NCEP: ATP III phenotypic criteria for metabolic syndrome were reinforced by measurements of Matsuda Index [21]. We have previously used the Matsuda index, which is considered a surrogate measure of whole body insulin sensitivity obtained from the OGTT [18,21]. However, in the present study, the Matsuda index was not solely used to determine groupings since a clear cut-off point for classification of insulin resistance versus insulin sensitivity in Hispanic populations has not yet been defined. In the present study, the metabolic syndrome criteria provides groupings that are reinforced by multiple variables, one of which being insulin sensitivity. Subjects classified with metabolic syndrome were more insulin resistant, which was fully anticipated since insulin resistance is thought to be the core defect underlying this condition [19,20]. It could be argued that the significant differences for the metabolic characteristics between the two groups could be due to age since the individuals without metabolic syndrome were slightly younger in age. 

Table S1 provided sex associated differences within the Met Syn-NO and Met Syn-YES groupings. The body fat percentage was significantly higher in women than men, and it has been shown that women tend to have larger proportion of body fat mass, and a higher yield of fat storage than men [31]. Fat metabolism involves the transportation of free fatty acids for storage. An important molecule which assists with this process is HDL. The levels of HDL in our subjects were significantly higher in women than men. The observed sex difference could be due to the premenopausal state in women, where there is typically lower total cholesterol and triglycerides, and higher HDL levels than men because of sex hormone regulation differences in fat metabolism [32]. This possible explanation could be applied to the current study based on the average female ages being below the typical age of menopause, and the same trend as described for the triglycerides, cholesterol, and HDL (as shown in Table S1). 

Another significant sex difference was found in the 2 hour plasma insulin concentrations, where the women were found to have higher levels than men. These results could be reflective of the study conducted by Magkos et al., which considered the effects of insulin and the suppression of endogenous glucose production between men and women [33]. When their subjects were infused with insulin to raise insulin concentrations to 15-20 mU/L, women had a prolonged suppression of endogenous glucose production than men [33]. This could infer that the proposed concentration of infused insulin is sustained longer in women. Another point of interest was the significantly higher levels of ALT in men than in women. A study performed elsewhere demonstrated that ALT (≥ 40 U/L) could be used as a predictor for coronary heart disease (CHD) in men, but not women [34]. The sex differences in ALT may also be due to the level of work performed by the liver in men (hepatic glucose production was not as easily suppressed) as opposed to women, as suggested in the aforementioned study [33]. Men were also found to present signs of CHD about ten years before women, so the average ages of the male and female subjects in our current study may add to the discrepancy [34].

In the present study, there was a higher expression of oxidative phosphorylation genes observed in the individuals classified with the metabolic syndrome phenotype. The higher expression in oxidative phosphorylation genes in whole blood is contradictory to the observed findings in skeletal muscle. We [15] and others [16] have demonstrated that nuclear encoded mitochondrial genes encoding proteins involved in electron transport and oxidative phosphorylation were decreased in insulin resistant muscle, and many of these changes could be attributed to lower peroxisome proliferator-activated receptor alpha (PPAR alpha) coactivator-1 alpha (PGC-1 alpha) gene expression [15]. We also showed that experimental insulin resistance produced by a lipid infusion in healthy volunteers decreases the mRNA expression of PGC-1 alpha and other mitochondrial and oxidative phosphorylation genes [28]. The discrepancies in the expression of genes involved in oxidative phosphorylation may be attributable to differences in tissue types and the role that each plays in the disease. A similar study using PAXgene tubes demonstrated an increased expression in oxidative phosphorylation genes in an obese Northern European White cohort; specifically cytochrome c oxidase subunits C6, B7, C7, and NADH dehydrogenase 1 alpha sub-complex 4 showed significant enrichment in both studies [30]. The increase in oxidative phosphorylation genes in whole blood may be caused by a response to increased energy demands in the metabolic syndrome individuals [30], or may be explained by an inflammation-induced increase in reactive oxygen species (ROS) generation. Although ROS was not measured in the present study, we know from other studies that in insulin resistant individuals the glucose levels in the bloodstream are elevated, which could increase the metabolic substrate input of the mitochondria leading to stress on the electron transport chain and causing an overproduction of ROS [35]. When the blood contains high amounts of ROS, a condition called oxidative stress is created. Previous studies have shown that oxidative stress plays key roles in diabetes, cardiovascular disease, and neurological disorders [36]. Although we observed similar findings to Ghosh et al., there were differences in our study design; our subjects were Latin descent, categorized based on metabolic syndrome criteria and we had much higher numbers of individuals entering into the analysis. 

The ribosome genes and pathway had a strong, distinct association with metabolic syndrome. Interestingly, all of the genes within the pathway showed an increase in expression. These results suggest that in the peripheral blood of individuals with the metabolic syndrome there is an increase in the cellular content of ribosomes augmenting the capacity for protein synthesis. The increase in ribosomes suggests that there is possibly an increase in protein synthesis to account for the increased energy demands observed in individuals with the metabolic syndrome; the significantly enriched genes possibly accounting for this phenomena (also found in Ghosh et al.) being ribosomal protein L7, S7, S24, and S31 [30]. Another study had shown a direct correlation between energy demands and ribosomal RNA synthesis through AMP-activated protein kinase in mammalian cells [37]. Their findings suggest that ribosomal RNA synthesis will increase when mitogenic conditions are optimal; for example, an overabundance of available nutrients. Therefore, it is possible that the physiological conditions of our sample population could provide optimal conditions for ribosomal RNA synthesis from the elevated levels of glucose. 

An increase in ribosomal genes could also be linked to inflammation. Insulin resistance causes an environment in which deoxyhypusine synthase (DHS) promotes cytokine-induced inflammation in β-cells, as well as catalyzes the conversion of lysine to hypusine that is unique to the translational elongation factor eIF5A found in the endoplasmic reticulum (ER) [38]. Therefore, when β-cell dysfunction occurs due to inflammatory stress, DHS also causes ER stress by eIF5A increased production of proteins [38]. A recent study performed by Yamaoka et al. on blood gene expression in Japanese cohorts separated by metabolic syndrome classifications also identified chronic low-grade inflammation related to ROS production; however, their study focused on data related to adipose tissue as opposed to skeletal muscle [39]. An interesting similarity between our studies is the increased expression of S100A8 related to metabolic syndrome explained by Yamaoka et al., which stated a correlation between the expression and ROS generation promoted by calprotectin (a S100A8/A9 complex) [40]. 

Our study demonstrated alterations in the MAPK signaling pathway genes with metabolic syndrome. Interestingly, all of the genes identified in the analysis were lower in expression in the metabolic syndrome individuals. We hypothesized that there would be alterations in genes involved in insulin signaling; however we expected to observe increased expression. Several studies have indicated that insulin resistance is connected to an escalation in activity from JNK and p38 in MAPK signaling pathway [41]. Since this was not the case in our study, the discrepancies may be explained by differences in blood tissue compared with skeletal muscle. The differences seen in blood may be related to the relationship between insulin resistance and endothelial dysfunction. Normally, insulin induces phosphatidylinositol 3-kinase dependent signaling (PI3K) to produce nitric oxide (NO) which increases blood flow in the vascular endothelium, and ultimately increases glucose uptake in skeletal muscle [42]. This process is accompanied by the MAPK-dependent insulin-signaling pathway that produces endothelin-1, which determines vascular responses to insulin [42]. Endothelial dysfunction is usually viewed as an imbalance that occurs in an insulin resistant state, where PI3K signaling is impaired and MAPK signaling is heightened [42]. The results from our study may indicate that blood is more sensitive to the effects occurring to PI3K in the vascular endothelium in relation to MAPK signaling, but not the actual enhanced effects of MAPK. 

In this study, the endocytosis pathway was lower in expression in the individuals classified with metabolic syndrome. These results are reinforced from a recent study on the role of autophagy in metabolism. Autophagy is a homeostatic mechanism that is typically known for transporting items such as damaged organelles or misfolded proteins to the lysosome to be degraded [43]. This process has also been viewed as a generator for cellular energy from protein degradation into amino acids and its role in the releasing free fatty acids to be used for beta-oxidation [43]. This ability to provide energy is increased under conditions of starvation coupled by internal stimuli such as oxidative stress, protein aggregation, and damaged organelles [44]. However, in our metabolic syndrome group, there is an excess supply of nutrients. Autophagy activity is decreased in environments with elevated glucose, because glucagon causes the up regulation in autophagy [43]. These findings provide some evidence with the decreased expression seen in endocytosis for our sample population. 

From our analysis a cluster of neurological pathways were found to be significant in metabolic syndrome, which included Alzheimer’s disease, Huntington’s disease, and Parkinson’s disease. The relevance of this finding is due to the growing efforts to study the late effects of metabolic syndrome type environments on the neurological system; mainly efforts to mark Alzheimer’s disease as type 3 diabetes [45]. The connection between metabolic syndrome and the nervous system is primarily due to insulin, which in the central nervous system plays a role in controlling neuronal function (memory and cognitive functioning), neurotransmitter release, and synaptic plasticity [45]. Insulin receptors are located in both neurons and glia of the brain, and are specifically prevalent in regions such as, the hypothalamus, cerebellum, olfactory bulb, hippocampus and cerebral cortex [46]. In conditions where prolonged elevation of glucose causes hyperinsulinemia (as associated with insulin resistance) in the bloodstream, a reduction of insulin crossing the blood brain barrier can occur [46]. Such an environment is thought to cause a shift to peripheral insulin use which can lead to downstream affects in memory, learning, and neuronal survival [45]. 

The systemic lupus erythematosus (SLE) pathway was found to be altered in our metabolic syndrome subjects. SLE has been described as a chronic inflammatory disease characterized by abnormal T and B cell function and production of antinuclear antibodies [47]. Inflammation is prominent in SLE, as well as insulin resistance and metabolic syndrome [2]. In the present study, an inflammatory marker that was increased in expression in the metabolic syndrome individuals was lactotransferrin, which has been shown to be increased in concentration during most inflammatory reactions [48]. Interestingly, in patients with SLE there is a four times greater chance of having the metabolic syndrome than without SLE [49]. Some of the genes affected in the oxidative phosphorylation pathway were also present in the SLE pathway. This could be attributed to the relationship of SLE with mitochondria. Individuals with SLE have been known to exhibit spontaneous apoptosis and defective activation-induced cell death of the T cells [47]. Since cell proliferation and apoptosis are energy-dependent processes, a disruption in the functionality of the mitochondria and alterations in ROS is suggested [47]. The most predominant finding in the SLE pathway analysis was the reduced expression in histone related genes in those individuals with metabolic syndrome. This decrease could be explained by a recent study that showed that the majority of individuals with SLE create anti-histone antibodies specifically directed towards histones within neutrophil extracellular traps (NETs) [49]. Neutrophils are one of the many cells found within whole blood, and if our population sample has similar physiology to SLE individuals it is possible that our results are showing that specific type of autoreactivity. 

Collectively, the global transcriptomic analyses on metabolic syndrome in our Latino population provided data that whole blood may be a useful surrogate for studying particular pathways. Based on our findings, pathways involved in ribosomes, Alzheimer’s disease, Huntington’s disease, Parkinson’s disease, systemic lupus erythematosus, and endocytosis may logistically continue studies using whole blood in relation to outcomes of metabolic syndrome. However, the pathways specifically linked to mitochondrial genes, such as oxidative phosphorylation and MAPK signaling have shown reversed outcomes in whole blood than seen in skeletal muscle for reasons that need further investigation.

## Supporting Information

Table S1 **Characteristics of subjects classified into one of two groups based on metabolic syndrome criteria and sex separated within those two groups.**
(PDF)

Table S2 **Results of KEGG exploratory pathway analysis performed in DAVID (http://david.abcc.ncifcrf.gov/) on the 1,432 genes that were altered in expression ≥ 1.2 fold and P < 0.05.**
(PDF)

Figure S1 **Oxidative phosphorylation genes with altered expression in individuals with metabolic syndrome.** The dashed line indicates no change in gene expression. All probes were altered in expression ≥ 1.2 fold and P < 0.05 (Benjamini corrected).
(PDF)

Figure S2 **MAPK signaling genes with altered expression in individuals with metabolic syndrome.** The dashed line indicates no change in gene expression. All probes were altered in expression ≥ 1.2 fold and P < 0.05 (Benjamini Hochberg corrected).
(PDF)
