# Supplementary material for: Whole Blood Gene Expression Profiles in Insulin Resistant Latinos with the Metabolic Syndrome
Source: PLoS One. 2013 Dec 17;8(12):e84002. doi: 10.1371/journal.pone.0084002 (PMC3866261; doi:10.1371/journal.pone.0084002)
Supplement: Table S1 — Characteristics of subjects classified into one of two groups based on metabolic syndrome criteria and sex separated within those two groups. (PDF) [file pone.0084002.s001.pdf]

Table S1 Characteristics of subjects classified into one of two groups based on metabolic syndrome criteria and sex separated within those two groups

	**Met Syn-NO**		**P Value Met Syn-NO**	**Met Syn-YES**		**P Value Met Syn-YES**
			**Male vs. Female**			**Male vs. Female**
**Gender**	Male (n=36)	Female (n=74)		Male (n=25)	Female (n=49)	
**Age (years)**	36.4±1.5	33.8±1.2	NS	36.5 ±2.3	40.5 ±1.7*	<0.05
**Body Mass Index (kg/m2)**	27.9±0.8	28.5±0.7	NS	36.2± 2.8[Ɨ]	34.5± 1.0[Ɨ]	NS
**Body Fat (%)**	21.9±1.0	32.3±0.7	<0.0001	28.3± 1.7*	37.7± 0.9[Ɨ]	<0.0001
**Systolic Blood Pressure (mmHg)**	120.6±2.1	111.8±1.1	<0.001	132.2± 3.6*	126.6±3.1[Ɨ]	NS
**Diastolic Blood Pressure (mmHg)**	78.0±1.3	74.0±0.9	<0.05	82.6± 2.1	81.0± 1.5[Ɨ]	NS
**Waist Circumference (cm)**	96.9±1.9	94.2±1.6	NS	113.2± 3.3[Ɨ]	107.0± 2.0[Ɨ]	<0.05
**Hip Circumference (cm)**	102.6±1.4	106.1±1.3	NS	113.1± 2.1[Ɨ]	117.8± 2.0[Ɨ]	NS
**Cholesterol**	172.8 ± 5.6	164.0 ± 3.4	NS	196.6 ± 7.8*	188.2 ± 5.9*	NS
**Triglyceride (mg/dL)**	122.7±9.1	100.3±4.3	<0.05	229.1± 22.4[Ɨ]	192.3± 14.3[Ɨ]	NS
**High Density Lipoprotein (mg/dL)**	41.3±1.5	50.9±1.3	<0.0001	33.4± 1.3**	39.7± 1.2[Ɨ]	<0.05
**Low Density Lipoprotein (mg/dL)**	111.1±4.6	98.1±2.9	<0.05	121.5± 8.7	115.4± 3.9*	NS
**Very Low Density Lipoprotein (mg/dL)**	20.5±1.5	16.8±0.7	<0.05	33.2± 2.8**	29.8± 1.7[Ɨ]	NS
**Alanine Aminotransferase (IU/L)**	29.8±3.0	19.5±1.1	<0.001	54.5± 6.5**	35.4± 4.0[Ɨ]	<0.05
**Aspartate Aminotransferase (IU/L)**	25.0±1.8	21.9±1.0	NS	34.6± 3.6*	29.0± 2.4*	NS
**Hemoglobin AIc (%)**	5.7 ±0.2	5.5 ±0.03	NS	6.0± 0.3	6.2± 0.2[Ɨ]	NS
**Fasting Plasma Glucose (mg/dL)**	98.2 ±5.1	89.9 ±1.0	<0.05	109.1± 5.8*	114.7± 6.6[Ɨ]	NS
**2 Hour Plasma Glucose (mg/dL)**	121.9 ±11.3	125.0 ±4.2	NS	154.1± 13.9*	196.0± 11.9[Ɨ]	<0.05
**Fasting Plasma Insulin (uIU/mL)**	6.5 ±0.6	7.8 ±0.6	NS	13.3± 2.0*	12.5± 1.7**	NS
**2 Hour Plasma Insulin (uIU/mL)**	34.5 ±4.9	73.3 ±7.3	<0.0001	77.0± 15.8*	124.3± 18.0**	<0.05
**Matsuda Index**	9.1 ±1.8	6.4 ±0.6	<0.05	3.3± 0.5[Ɨ]	3.7± 0.6[Ɨ]	NS

Data are mean ± SE
Significance of <0.05 indicated by *, <0.001 by **, and <0.0001 by [Ɨ] within sex for Met Syn-YES versus NO.
