# Supplementary material for: Whole Blood Gene Expression Profiles in Insulin Resistant Latinos with the Metabolic Syndrome
Source: PLoS One. 2013 Dec 17;8(12):e84002. doi: 10.1371/journal.pone.0084002 (PMC3866261; doi:10.1371/journal.pone.0084002)
Supplement: Table S2 — Results of KEGG exploratory pathway analysis performed in DAVID (http://david.abcc.ncifcrf.gov/) on the 1,432 genes that were altered in expression ≥ 1.2 fold and P < 0.05. (PDF) [file pone.0084002.s002.pdf]

Table S2 Results of KEGG exploratory pathway analysis performed in DAVID (http://david.abcc.ncifcrf.gov/) on the 1,432 genes that were altered in expression ≥ 1.2 fold and P < 0.05

KEGG pathway	Probe Names	Fold Enrichment	Benjamini
hsa03010:Ribosome (n=119)	A_24_P938169, A_32_P158746, A_32_P86728, A_23_P7066, A_23_P33045, A_32_P22078, A_32_P92295, A_32_P220127, A_32_P208178, A_32_P118258, A_23_P69431, A_23_P2725, A_32_P72940, A_32_P182882, A_24_P67100, A_24_P232856, A_24_P161604, A_24_P41160, A_32_P25371, A_24_P247153, A_32_P20367, A_24_P357518, A_23_P44956, A_32_P146508, A_32_P186981, A_24_P403403, A_24_P830667, A_32_P21384, A_24_P417606, A_24_P20777, A_24_P106306, A_23_P144497, A_32_P31182, A_32_P24581, A_32_P135818, A_32_P11931, A_24_P418418, A_32_P16747, A_32_P136319, A_23_P318646, A_24_P375237, A_32_P205553, A_32_P30710, A_23_P204472, A_32_P234738, A_24_P383999, A_24_P303118, A_24_P203909, A_23_P76961, A_32_P113742, A_24_P188878, A_23_P44974, A_32_P196483, A_32_P220307, A_23_P128067, A_23_P217609, A_24_P159036, A_32_P14744, A_24_P213354, A_23_P112429, A_32_P155364, A_23_P214666, A_23_P120660, A_24_P29818, A_32_P145153, A_24_P606663, A_24_P850428, A_32_P93782, A_24_P280803, A_32_P57237, A_24_P341677, A_23_P26713, A_24_P280897, A_23_P74629, A_23_P65768, A_24_P418619, A_23_P90143, A_23_P34018, A_32_P219657, A_32_P215714, A_24_P548354, A_24_P358164, A_23_P7221, A_23_P14734, A_24_P213783, A_24_P349743, A_32_P164630, A_32_P203154, A_23_P151321, A_32_P202488, A_23_P117721, A_32_P14894, A_32_P58074, A_32_P194821, A_23_P123330, A_23_P1206, A_24_P272061, A_23_P12140, A_32_P219031, A_32_P84605, A_32_P11477, A_32_P235128, A_24_P50554, A_32_P184518, A_23_P141549, A_24_P126890, A_24_P50157, A_24_P41180, A_24_P221366, A_24_P315184, A_24_P169378, A_24_P148235,	8.3	<0.0001

	A_24_P280901, A_23_P256933, A_23_P62605, A_32_P175580, A_24_P144025, A_23_P143958, A_23_P141389		
hsa00190:Oxidative phosphorylation (n=32)	A_23_P91769, A_23_P56314, A_23_P159833, A_24_P343377, A_24_P348649, A_24_P102769, A_23_P140423, A_23_P110811, A_23_P8900, A_24_P302998, A_23_P137814, A_23_P63655, A_24_P96527, A_23_P159650, A_24_P23245, A_23_P252322, A_24_P333733, A_23_P154832, A_23_P81690, A_32_P98313, A_23_P215832, A_24_P14434, A_23_P213718, A_23_P20882, A_23_P125420, A_23_P10463, A_32_P123771, A_23_P75369, A_23_P31844, A_23_P51009, A_24_P406301, A_23_P145777	3.0	<0.0001
hsa05010:Alzheimer disease (n=31)	A_23_P91769, A_23_P56314, A_23_P159833, A_24_P348649, A_24_P102769, A_23_P140423, A_23_P110811, A_24_P111342, A_23_P8900, A_23_P92623, A_23_P63655, A_23_P159650, A_24_P23245, A_23_P252322, A_23_P154832, A_24_P364363, A_23_P81690, A_32_P98313, A_24_P14434, A_23_P213718, A_23_P139722, A_23_P4944, A_32_P228124, A_23_P10463, A_23_P112801, A_23_P79518, A_32_P123771, A_23_P34402, A_23_P51009, A_24_P406301, A_23_P145777	2.4	0.0038
hsa05120:Epithelial cell signaling (n=15)	A_23_P135755, A_24_P333733, A_24_P319364, A_23_P106002, A_23_P159920, A_23_P147431, A_23_P20882, A_32_P6344, A_23_P67932, A_23_P75369, A_23_P31844, A_23_P137814, A_23_P426292, A_23_P7144, A_23_P75989	3.3	0.0051
hsa05322:Systemic lupus erythematosus (n=31)	A_23_P93258, A_24_P91035, A_24_P174924, A_23_P30776, A_23_P111054, A_23_P42178, A_23_P42198, A_32_P12214, A_23_P200728, A_23_P59069, A_23_P133814, A_24_P323835, A_24_P45651, A_23_P103981, A_23_P395374, A_23_P70445, A_23_P59045, A_23_P152516, A_24_P148321, A_24_P7934, A_24_P82419, A_24_P9321, A_23_P219045, A_23_P111037, A_23_P167983, A_24_P590537, A_23_P23141, A_23_P105957, A_23_P366216, A_24_P938284, A_23_P8013	2.5	0.0223
hsa05016:Huntington disease (n=29)	A_23_P91769, A_23_P163850, A_23_P134176, A_23_P56314, A_23_P159833, A_24_P348649, A_24_P102769, A_23_P157452, A_23_P140423, A_23_P110811, A_24_P111342, A_23_P8900,	2.1	0.0252

	A_23_P63655, A_23_P159650, A_24_P23245, A_23_P154832, A_23_P252322, A_23_P81690, A_32_P98313, A_24_P14434, A_23_P213718, A_23_P382775, A_32_P228124, A_23_P157117, A_23_P10463, A_32_P123771, A_23_P51009, A_24_P406301, A_23_P145777		
hsa05012:Parkinson disease (n=23)	A_23_P252322, A_23_P154832, A_23_P91769, A_23_P81690, A_23_P56314, A_32_P98313, A_23_P159833, A_24_P14434, A_23_P213718, A_24_P348649, A_24_P102769, A_23_P140423, A_23_P110811, A_23_P10463, A_24_P111342, A_23_P8900, A_32_P123771, A_23_P51009, A_24_P406301, A_23_P63655, A_23_P145777, A_24_P23245, A_23_P159650	2.2	0.0454
hsa04144:Endocytosis (n=28)	A_23_P167389, A_24_P398432, A_24_P912382, A_23_P337242, A_23_P105184, A_23_P52647, A_23_P95917, A_23_P391198, A_23_P67932, A_23_P31873, A_23_P114903, A_23_P47517, A_24_P123616, A_23_P135755, A_24_P682285, A_23_P141688, A_23_P202334, A_23_P417282, A_23_P153236, A_23_P60816, A_23_P27023, A_23_P158829, A_23_P145264, A_23_P339191, A_23_P55551, A_23_P102000, A_23_P111132, A_23_P335848	1.9	0.0502
hsa04010:MAPK signaling pathway (n=36)	A_23_P313640, A_23_P79398, A_23_P41796, A_23_P337242, A_32_P104075, A_24_P304636, A_23_P37375, A_24_P94054, A_23_P218774, A_23_P114903, A_23_P139704, A_24_P935026, A_23_P92623, A_23_P98275, A_24_P123616, A_23_P75989, A_24_P396650, A_23_P77847, A_24_P364363, A_24_P682285, A_23_P110712, A_23_P202334, A_23_P159920, A_23_P139722, A_24_P346277, A_32_P6344, A_23_P106194, A_23_P501933, A_23_P112801, A_23_P207131, A_23_P79518, A_23_P40952, A_23_P158829, A_23_P111132, A_23_P142310, A_23_P426292	1.7	0.0571
hsa04662:B cell receptor signaling pathway (n=13)	A_23_P77847, A_23_P106002, A_23_P147431, A_23_P159920, A_32_P70158, A_23_P218774, A_23_P106194, A_23_P368711, A_23_P112801, A_23_P9255, A_23_P40952, A_23_P92623, A_24_P938284	2.4	NS
hsa04062:Chemokine signaling pathway	A_23_P51534, A_23_P135755, A_23_P250302, A_23_P77847, A_23_P106002, A_23_P147431, A_23_P159920, A_32_P116203,	1.7	NS

(n=22)	A_23_P218774, A_23_P413641, A_23_P67932, A_24_P184555, A_24_P71021, A_23_P121596, A_24_P185394, A_23_P40952, A_23_P158829, A_23_P102000, A_24_P148717, A_24_P365526, A_23_P7144, A_23_P75989		
hsa03040:Spliceosome (n=21)	A_23_P165402, A_23_P253571, A_23_P421563, A_24_P682285, A_23_P104676, A_23_P200216, A_24_P263543, A_23_P127233, A_23_P84775, A_24_P226248, A_23_P93750, A_23_P114903, A_23_P141636, A_23_P132863, A_24_P29001, A_23_P126291, A_23_P116016, A_23_P111132, A_23_P16132, A_24_P74160, A_24_P123616	1.9	NS
hsa04666:Fc gamma R-mediated phagocytosis (n=13)	A_23_P147431, A_32_P116203, A_23_P218774, A_23_P200728, A_23_P20196, A_23_P9255, A_23_P211445, A_23_P40952, A_23_P214222, A_23_P203743, A_24_P938284, A_24_P365526, A_23_P75989	2.0	NS
hsa05215:Prostate cancer (n=13)	A_23_P77847, A_23_P163850, A_23_P106002, A_23_P202334, A_23_P417282, A_23_P159920, A_24_P222599, A_23_P157117, A_24_P111342, A_32_P199252, A_23_P40952, A_23_P385034, A_23_P162866	2.0	NS
hsa03050:Proteasome (n=9)	A_32_P43217, A_23_P345591, A_23_P25735, A_24_P124992, A_23_P140301, A_23_P65254, A_23_P42664, A_23_P304287, A_23_P79628	2.5	NS
