# Supplementary material for: Whole Blood Gene Expression Profiles in Insulin Resistant Latinos with the Metabolic Syndrome
Source: PLoS One. 2013 Dec 17;8(12):e84002. doi: 10.1371/journal.pone.0084002 (PMC3866261; doi:10.1371/journal.pone.0084002)

Figure S2 MAPK signaling genes with altered expression in individuals with metabolic syndrome. The dashed line indicates no change in gene expression. All probes were altered in expression ≥ 1.2 fold and $P < 0.05$ (Benjamini Hochberg corrected)

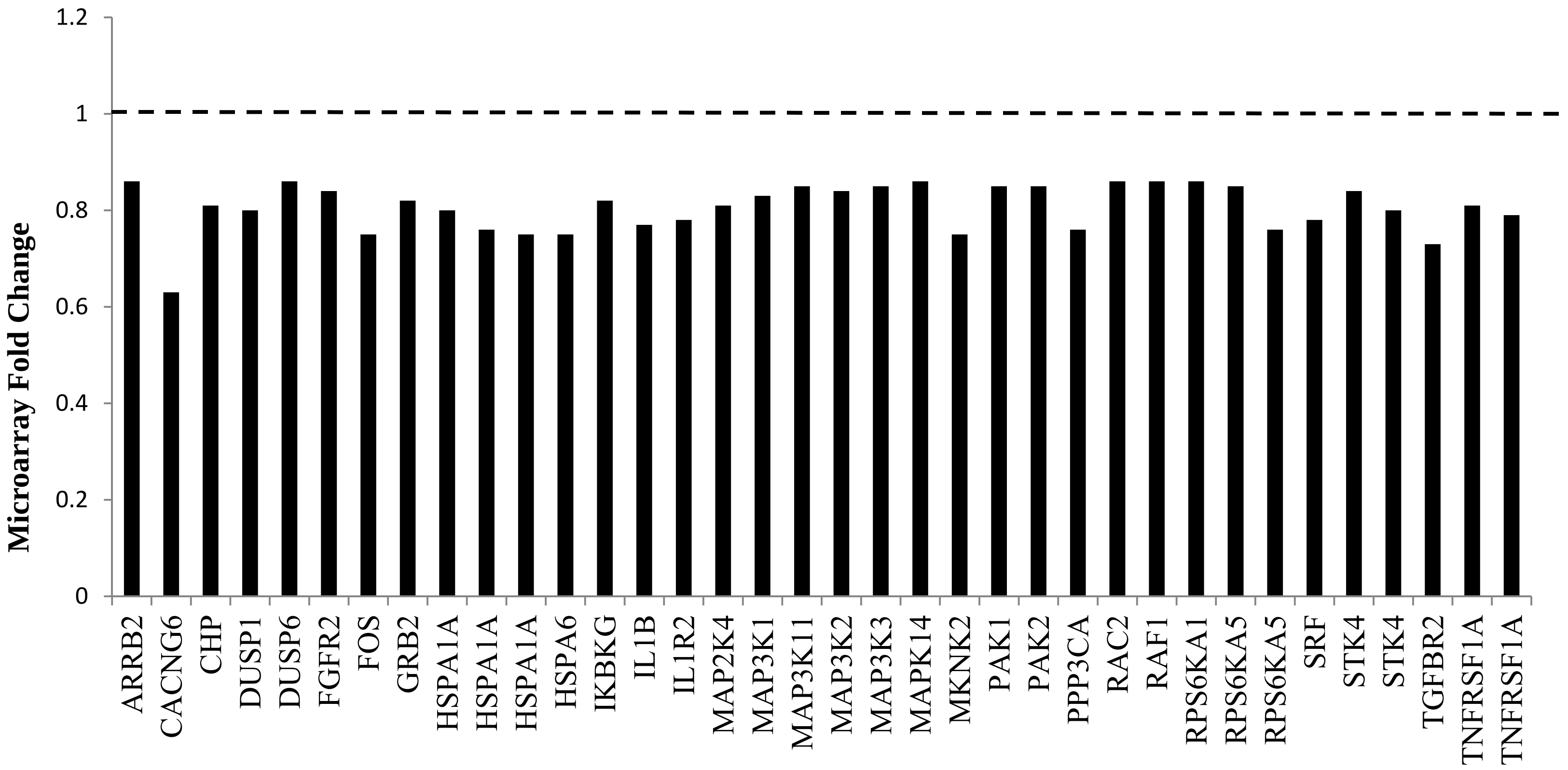

Supplement: Figure S2 — MAPK signaling genes with altered expression in individuals with metabolic syndrome. The dashed line indicates no change in gene expression. All probes were altered in expression ≥ 1.2 fold and P < 0.05 (Benjamini Hochberg corrected). (PDF) [file pone.0084002.s004.pdf]
